# Beyond monoclonal gammopathy of undetermined significance, clinical spectrum of immunoglobulin M gammopathy: a case series with focus on the diagnostic and management challenges

**DOI:** 10.2217/ijh-2022-0006

**Published:** 2023-02-24

**Authors:** Omer S Ashruf, Saeid Mirzai, Laeth L George, Faiz Anwer

**Affiliations:** 1 Northeast Ohio Medical University, Rootstown, OH 44139, USA; 2 Department of Internal Medicine, Cleveland Clinic, Cleveland, OH 44195, USA; 3 Division of Hematology & Oncology, Cleveland Clinic, Cleveland, OH 44195, USA

**Keywords:** Bing–Neel syndrome, cryoglobulinemia, IgM gammopathy, lymphoplasmacytic lymphoma, plasma cell dyscrasia, Waldenström macroglobulinemia

## Abstract

Immunoglobulin M monoclonal gammopathy is detected in Waldenström macroglobulinemia (WM), a rare lymphoplasmacytic lymphoma with serum immunoglobulin M. We report three rare presentations with focus on diagnostic and management challenges of type I cryoglobulinemia, type II cryoglobulinemia, and Bing–Neel syndrome. In approximately 10% of WM cases, macroglobulins can precipitate to cryoglobulins. Type I and II cryoglobulinemia, representing 10–15% and 50–60% of WM cases, respectively, present with vasculitis and renal failure. Bing–Neel syndrome, representing 1% of WM patients, is a rare neurological complication with lymphoplasmacytic infiltration in the brain. WM diagnosis includes bone marrow biopsy, immunophenotypic analysis, and *MYD88* L265P mutation. We initiated management of cryoglobulinemia with dexamethasone, rituximab, and cyclophosphamide; in Bing–Neel, bortezomib and dexamethasone, followed by a Bruton tyrosine kinase inhibitor.

## Plain language summary

Lymphoplasmacytic lymphoma (LPL) is an aberrant proliferation of plasma cells which may present as Waldenström macroglobulinemia, a disease characterized by high levels of immunoglobulin M that may result in deposition in bone marrow, spleen, and lymph nodes. The current understanding of clinical presentation is limited: patients with LPL may present with a wide range of symptoms related to paraproteinemia or tumor infiltration. This case series elucidates on specific and rare subsets of LPL, namely types I and II cryoglobulinemia and Bing–Neel syndrome. This report showcases the uncommon symptomatology of immunoglobulin M kappa deposition, such as kidney failure and neurological defects. The diagnostic and management challenges are of specific interest in this report, considering criteria, such as bone marrow biopsy, immunofixation, and cerebrospinal analysis. Literature on treatment protocols is equally limited and this report considers dexamethasone-rituximab-cyclophosphamide protocol and Bruton tyrosine kinase inhibitors compared to other common regimens. This report can be of great use to clinical oncologists by adding to the working knowledge on the rare manifestations of LPL and how to approach diagnostic and management challenges.

## Tweetable abstract

Case series showcases diagnostic and management criteria of three diverse presentations of IgM gammopathy: type I cryoglobulinemia, type II cryoglobulinemia, and Bing–Neel syndrome.

**Practice points**

- The current literature on the rare manifestations of Waldenström macroglobulinemia (WM) is limited; research on diagnostic criteria and management is equally scarce.
- WM, a subset of lymphoplasmacytic lymphoma, is characterized by elevated levels of serum immunoglobulin M monoclonal protein.
- WM presents with a broad constellation of symptoms, ranging from fatigue, weight loss, and fever to renal failure, neurological deficits, and vasculitis.
- This report focuses on niche manifestations of WM, namely type I cryoglobulinemia, type II cryoglobulinemia, and Bing–Neel syndrome.
- Approximately 91% of cases are identified by a leucine-to-proline somatic mutation on the *MYD88* gene at position 265.
- Other diagnostic criteria include bone marrow biopsy, immunophenotyping, cryocrit level in cryoglobulinemia, and cerebrospinal fluid analysis in Bing–Neel syndrome.
- Type I cryoglobulinemia often presents with a greater than 10% cryocrit level, as cryoglobulins have a predilection to precipitate in serum. However, cryocrit levels may do not detect type II cryoglobulinemia due to its mixed nature, and immunofixation was the preferred diagnostic tool.
- Although many major classes of chemotherapeutics are effective in managing WM, dexamethasone, rituximab, and cyclophosphamide (DRC) was efficacious in the type I and II cryoglobulinemia cases, showing an 80% survival rate in the literature and minimal toxicities compared with other common regimens.
- Bortezomib and pulsed dexamethasone for 4 days with initiation of a Bruton tyrosine kinase inhibitor outpatient was efficacious in the Bing–Neel case and enhanced central nervous system penetration.

Immunoglobulin M (IgM) monoclonal gammopathy can present as a manifestation of plasma cell disorders or can be associated with lymphoproliferative disorders. Lymphoplasmacytic lymphoma (LPL), a rare B-cell lymphoma in which clonal cells can involve the bone marrow and, occasionally, spleen and lymph nodes, is often associated with WM. WM involves elevated serum IgM monoclonal protein along with LPL and its reported incidence is three cases per million people [1]. Patients with WM broadly present with constitutional “B symptoms” such as fatigue, weight loss, and fever in addition to organomegaly (most commonly lymphadenopathy and hepatosplenomegaly) and hyperviscosity syndrome. The temperature-sensitive nature of IgM can also lead to precipitation and deposition, giving rise to detectable cryoglobulins and thus presentation with cryoglobulinemia [2]. Diagnostic confirmation of WM/LPL can come from detecting the L265P amino acid substitution in *MYD88*, which is seen in most cases. Here, we discuss three cases of educational value indicative of the varied spectrum of cryoglobulinemia, in addition to the genetic findings of WM, the diagnosis criteria, management protocols, and a rare manifestation of LPL/WM known as Bing–Neel syndrome (Table 1).

**Table 1.** A summary of laboratory results of the three cases: type I cryoglobulinemia, type II cryoglobulinemia, and Bing–Neel syndrome.

Test	Case 1	Case 2	Case 3	Reference
Hemoglobin	9.9	8.1	13.7	11.5–15.5 g/dl
White blood cells	3.17	6.63	7.07	3.70–11.00 k/ul
Neutrophils	51.1	75.0	58.8	
Lymphocytes	32.5	16.4	31.2	
Platelets	229	201	261	150–400 k/ul
Creatinine	1.36	1.73	0.73	0.58–0.96 mg/dl
Blood urea nitrogen	24	41	12	7–21 mg/dl
Sodium	139	135	126	136–144 mmol/l
Total bilirubin	0.7	0.2	0.7	0.2–1.3 mg/dl
Alkaline phosphatase	52	128	79	34–123 U/l
Alanine amino transferase	11	8	37	7–38 U/l
Calcium	10.4	8.0	9.0	8.5–10.2 mg/dl
Phosphorus	4.4	2.8	3.8	2.7–4.8 mg/dl
Albumin	4.2	2.8	3.7	3.9–4.9 g/dl
Lactate dehydrogenase	201	159	229	135–214 U/l
Urine microscopy	Few granular casts, 10–20 normomorphic RBCs, 5–10 WBCs	Few granular casts, 3–5 normomorphic red blood cells, 3–5 white blood cells		Casts are per low-power field, cells are per high-power field
Urine protein-to-creatinine ratio	1.5	5.5		<0.2
Serum protein electrophoresis interpretation	M protein identified	M protein identified	M protein identified	
M protein location	Gamma fraction	Gamma fraction	Gamma fraction	
M protein concentration	0.93	0.09 and 0.21	0.18	0.00 gm/dl
Serum kappa to lambda ratio	5.91	3.10	0.93	0.26–1.65
Complement 3	53	54		86–166 mg/dl
Complement 4	<2	<2		13–46 mg/dl
Anti-nuclear antibodies	1:640	>1:1,280		Negative
Anti-nuclear antibody pattern	Speckled	Centromere		
SSA antibody	Positive	Positive		Negative
SSB antibody	Positive	Positive		Negative
Rheumatoid factor	56			<16 IU/ml
Cryoglobulin, qualitative	Negative	Positive	Negative	Negative
IgA cryoprecipitate		3		0 mg/dl
IgG cryoprecipitate		17		0 mg/dl
IgM cryoprecipitate		26		0 mg/dl
Human immunodeficiency virus 1/2	Negative	Negative	Negative	Negative
Hepatitis B surface antigen	Negative	Negative		Negative
Hepatitis B surface antibody	Negative	Negative		Negative
Hepatitis B core antibody	Negative	Negative		Negative
Hepatitis C antibody	Negative	Negative		Negative

## Case reports

### Case 1

A 64-year-old female with a past medical history significant for rheumatoid arthritis, Sjögren's syndrome, and osteoporosis lost to follow-up by rheumatology presented to the family medicine clinic for a routine checkup. Physical exam was significant for hyperpigmented lesions along the bilateral anterior shins with sparse purpura and bilateral ulnar deviation of metatarsophalangeal joints. Routine labs were performed showing known leukopenia, new normocytic anemia, new renal failure (baseline of 0.8 mg/dl 2 years prior), hypercalcemia, and new hypothyroidism (Table 1). She was referred to hematology, where workup was significant for an IgM kappa monoclonal gammopathy based on serum protein electrophoresis (SPEP). A bone marrow biopsy was negative without any lymphoproliferative process.

Her anemia continued to worsen, with intermittent need for transfusions. A urine dipstick indicated nephrotic-range proteinuria. With the remainder of her presentation and history, this was concerning for monoclonal gammopathy of renal significance. She was admitted to the hospital for kidney biopsy, which showed proliferative glomerulonephritis and vasculitis with IgM kappa deposition, suggestive of type I cryoglobulinemia (Table 2). Computed tomography (CT) of the chest, abdomen, and pelvis showed mild diffuse lymphadenopathy; biopsy of axillary lymph nodes was negative. Chemotherapy was initiated with rituximab, cyclophosphamide, and dexamethasone which she tolerated well. She was discharged home with improved kidney function and plan to continue with six total cycles of chemotherapy. Following tumor board discussion, the decision was made to continue rituximab (during the first and fourth month of treatment), cyclophosphamide, and dexamethasone with the addition of bortezomib. Patient completed the treatments with improvement in kidney function and blood counts. Labs at 1-year follow-up showed M protein concentration of 0.24 gm/dl and serum kappa to lambda ratio 1.24.

**Table 2.** Formal diagnostic criteria of type I cryoglobulinemia, type II cryoglobulinemia, and Bing–Neel syndrome.

	Case 1: type I cryoglobulinemia	Case 2: type II cryoglobulinemia	Case 3: Bing–Neel syndrome
Diagnostic Criteria	○ Cryocrit level (>10%) ○ Immunochemical analysis/immunofixation (IgM heavy chains) ○ Biopsy (immune complex vasculitis)	○ Immunochemical analysis/immunofixation (polyclonal IgG chains) ○ Cryocrit level (2–7%) ○ Biopsy (immune complex vasculitis)	○ Cerebral spinal fluid analysis (clonal B cells) ○ MRI of CNS (leptomeningeal or parenchymal involvement)

### Case 2

A 67-year-old female, with a past medical history of Sjögren's syndrome with interstitial lung disease, heart failure with preserved ejection fraction, bicuspid aortic valve with mild stenosis, poorly-controlled hypertension, hyperlipidemia, and irritable bowel syndrome presented to our facility via transfer for altered mental status, uncontrolled hypertensive urgency, acute abdominal pain, and anasarca. She had been in her usual state of health until 1 month prior, when she was hospitalized with acute renal failure (baseline of 1.0 mg/dl 2 months earlier), microcytic anemia, and acute on chronic diastolic heart failure (Table 1). She received adequate diuresis, intravenous iron, and antibiotics for possible urinary tract infection and pneumonia. The patient also complained of right leg neuropathy, which she attributed to a herpes zoster infection several months prior. Recent workup at the outside hospital was significant for a skeletal x-ray survey with lucent foci of the calvarium, humeri, femur, and right pubic ramus with elevated serum free kappa to lambda light chain ratio (3.57, normal 0.26–1.65), but SPEP, cytogenetics, flow cytometry, and FISH were negative. Bone marrow biopsy was normocellular with negative Congo Red staining. Abdominal fat pad biopsy and cardiac MRI were performed and negative for amyloidosis.

At our facility, CT of the head was negative, and electroencephalogram indicated severe diffuse encephalopathy, likely toxic-metabolic. Mental status returned to baseline following hypertensive management. CT of the abdomen and pelvis showed anasarca, ileum wall thickening, and mesenteric edema with arterial atherosclerosis. Lactate was within normal limits, and infectious diarrhea workup was negative. Colonoscopy with ileum and colon biopsy did not demonstrate amyloid deposition or Whipple disease, and diarrhea improved with anti-diarrheals. Urine protein-to-creatinine ratio showed severe proteinuria, and M protein was identified on serum (two M proteins in serum). Urine protein electrophoresis with immunofixation showed an IgG kappa monoclonal gammopathy. Renal biopsy was performed showing diffuse proliferative glomerulonephritis with IgM kappa deposition, moderate arterio- and arteriolosclerosis, and moderate tubular atrophy and interstitial fibrosis. Congo Red stain was negative for amyloid. Following cryoglobulin detection in serum, immunofixation characterized the disorder as a type II cryoglobulinemia with monoclonal IgM kappa and polyclonal IgG (Table 2).

Whole-body PET was obtained without any focal neoplastic process identified. The final diagnosis was attributed to WM with IgM kappa disease, type II cryoglobulinemia involving the kidneys. Treatment was initiated inpatient with dexamethasone, rituximab, and cyclophosphamide which she tolerated well. Medical management was also initiated for her glomerulonephritis, and follow-up was scheduled with outpatient nephrology. She was discharged home in stable condition. The treatment was continued outpatient and she had improvement in kidney function and proteinuria (urine protein-to-creatinine ratio 0.4) after three cycles with significant improvement in edema and hypertension at 3-month follow-up.

### Case 3

A 73-year-old male with a past medical history of hypertension, hyperlipidemia, recent right lower extremity deep vein thrombosis, and gout presented to our hospital for a second opinion regarding intermittent waxing and waning focal neurologic deficits which began ten months prior after being bitten by a deer tick while hiking. The symptoms started as right foot weakness, which later progressed to foot drop with paresthesia up to the knee. This was followed by left facial droop, treated as Bell's Palsy, followed by diplopia and left ptosis. Subsequently, he had right facial droop and ptosis, and he was started on steroid taper and pyridostigmine without improvement. He additionally had intermittent ataxia, weakness, falls, dysarthria, dysphonia, dysphagia, arthralgia, and leg swelling. Physical exam was significant for right facial droop with facial diplegia and significant lower extremity weakness and decreased sensation (right worse than left) but intact reflexes. He had undergone extensive testing at outside hospitals, which were significant for IgM kappa monoclonal gammopathy, axonal sensorimotor polyneuropathy on electromyography (EMG), and lumbar puncture with elevated IgG and albumin (normal IgG/albumin) and negative infectious testing. Tick-borne illness, myasthenia gravis, and autoimmune testing were negative.

Routine labs were significant for chronic hyponatremia but otherwise unremarkable (Table 1). The previous M protein was detected without renal dysfunction, anemia, lymphadenopathy, hepatosplenomegaly, or bone involvement. MRI of the brain and spine was performed, showing extensive thickening and enhancement of his cranial nerves and lumbosacral roots (Table 2). EMG showed asymmetrical axonal sensorimotor polyradiculoneuropathy. CT of the chest, abdomen, and pelvis and whole-body PET was without signs of malignancy or metastasis. CT angiography of the head showed severe right V4 vertebral artery and basilar artery multifocal tapering stenosis without infarction. Serum interleukin-2 receptor and Ganglioside GM2 antibodies were positive, but likely from known neuronal damage seen on EMG. Myelin-associated glycoprotein and vascular endothelial growth factor were negative. Urine protein electrophoresis was negative for M protein, but SPEP with immunofixation confirmed IgM kappa monoclonal gammopathy.

Repeat lumbar puncture was performed with studies showing pleocytosis (17/ul, normal 0–5/ul), elevated protein (219 mg/dl, normal 15–45 mg/dl), and elevated CNS IgG, IgG index, and IgG synthesis. Interestingly, flow cytometry showed lymphocytes with a mixture of 83% CD5^+^ T-cells and 10% monotypic B cells; the B cells expressed CD19, CD20, CD45, and kappa light chain, compatible with B-cell lymphoproliferative disorder, likely LPL given the IgM kappa monoclonal gammopathy. A bone marrow biopsy showed lambda monotypic B cells with CD5 and CD23 positivity and atypical lymphoid infiltrate involving approximately 5–10% of cellularity, inconsistent with the CSF findings. However, the CSF findings, the thickening and enhancement of the lumbosacral nerve roots on MRI, and multiple CNS symptoms established the diagnosis of Bing–Neel syndrome (Table 2).

Treatment was started inpatient with one dose of bortezomib and pulsed high-dose dexamethasone for four days with the plan to continue therapy outpatient with ibrutinib, a Bruton tyrosine kinase (BTK) inhibitor with CNS penetration. Intrathecal therapy was felt to not be necessary with the treatment plan. The patient was discharged to acute rehab in stable condition. He had complete hematologic remission with this therapy, with labs at the 1-year follow-up showing no definitive M protein. Furthermore, he had improvement in his lower extremity weakness and facial nerve dysfunction, allowing for the gradual regaining of independence and ambulation without an assistive device.

## Discussion

This case series highlights the complexity of diagnosis of IgM monoclonal gammopathy related disorders and spectrum of its presentations. Type I cryoglobulinemia is a monoclonal gammopathy of IgM deposition (or IgG and, less commonly, IgA), which typically manifests as systemic vasculitis. It may also present as skin lesions and progress to renal dysfunction and neurological deficits [3]. The underlying diseases to cause Type I cryoglobulinemia include a range of lymphoproliferative disorders such as multiple myeloma and less commonly monoclonal gammopathy of undetermined significance [4,5]. Cryocrit level, a measure of cryoglobulin precipitate in total serum, is a diagnostic criterion that is generally elevated in Type I, often greater than 10% (Table 2) [6,7].

Type II cryoglobulinemia presents with mixed cryoglobulins with a composition of monoclonal IgM and polyclonal IgG. Clinical presentation may be nonspecific, but purpura is seen in approximately 90% of patients and other skin manifestations in 69 to 86% of patients [8]. The pathological etiology in 80–90% of mixed cryoglobulinemic cases is hepatitis C virus, and also associated with autoimmune diseases [9]. Determining cryocrit levels and immunochemical typing are used as diagnostic criteria [10], although mixed cryoglobulinemia can have low cryocrit levels (Table 2) [6]. Bing–Neel syndrome is a rare presentation of WM secondary to malignant lymphoplasmacytic cell infiltration of the central nervous system and has a diverse clinical picture including a wide range of cognitive defects [11]. Ataxia, facial and oculomotor cranial nerve involvement are detected [12]. Bing–Neel can present in two distinct ways, the more common of which presents diffusely and with leptomeningeal involvement with LPL, but is also seen in tumor form that presents in the subcortical regions [13]. As a result, the strongest diagnostic tools are CSF analysis, MRI, and/or histological biopsy of the cerebrum or meninges, which would indicate infiltration (Table 2). The pan B-cell profile expresses antigens for CD19, CD20, CD79a, and CD79b [13]. In case three, B-cells expressed CD19, CD20, CD45, and kappa light chain. Further, case three was positive for myeloma protein (M protein) in SPEP (along with cases one and two), an abnormal protein produced by plasma cells and an essential component of Bing–Neel syndrome diagnostic workup [14], confirming plasma cell proliferation.

WM is characterized by a somatic missense mutation in amino acid configuration (L265P) of the *MYD88* gene in 91% of LPL cases [15]. This mutation is conferred by chromosomal abnormalities, namely 6q21-25 deletions, which are implicated in 40–50% of patients and inactivate key regulatory factors in cell signaling [16]. Although the mechanism of action is unclear, *MYD88* binds to BTK, enhancing phosphorylation and augmenting tumor survival [17]. The leucine-to-proline amino acid substitution of *MYD88* causes activation of signaling cascade *NF-κB* which enhances cell proliferation and is of clinical significance [18]. Somatic *CXCR4* mutation is seen in patients with WM although not as ubiquitous as *MYD88* mutation [19].

Although renal insufficiency is seen in approximately 3% of patients with WM [20], cases one and two had a renal biopsy performed, which indicated diffuse proliferative glomerulonephritis with IgM kappa deposition. IgM deposition and immune complexes show in the basement membrane of the glomerulus, leading to nephrotic range proteinuria [21]. Case two was diagnosed as type II cryoglobulinemia with monoclonal IgM kappa and polyclonal IgG detection via immunofixation. Due to the rare nature of the disease, there is a scarcity of comparative literature on WM treatments, making treatment selection especially difficult. Of the literature reviewed, single-agent rituximab is a less effective treatment option, with a 13% response rate in patients with serum IgM levels above 4000 mg/dl [22]. In select cases, single-agent rituximab can cause an increase in IgM levels, further exacerbating hyperviscosity symptoms [22]. However, rituximab plays a central role in many initial treatment protocols for WM patients. In patients with low disease burden and a need for rapid disease control, dexamethasone-rituximab-cyclophosphamide (DRC) protocol is recommended [23]. A clinical trial found in patients who responded to the DRC protocol, the two-year survival rate was 80% [24]. DRC may also present with fewer toxicities associated with other common protocols (bendamustine + rituximab), with 9% of patients showing grade 3 and 4 toxic effects [25]. Cases 1 and 2 (type I and II cryoglobulinemia, respectively) were initiated on DRC.

Although there is overlap in pathogenesis between monoclonal gammopathy of undetermined significance (MGUS) and WM, such as the presence of plasma cell dyscrasia and subsequent IgM paraproteinemia, WM is distinct in its diagnostic criteria and clinical presentation. A defining characteristic, WM demonstrates greater than 10% monoclonal lymphoplasmacytic cell infiltrate in bone marrow, whereas MGUS has shown less than 10% [26]. MGUS, a generally asymptomatic disorder [27], progresses to MM in 5–7% of cases but characteristics of MGUS consistently precede MM [28]. WM, however, presents with hyperviscosity-associated symptoms, lymphadenopathy and hepatosplenomegaly not seen in MGUS.

Other common associations with WM include Schnitzler syndrome and anti-myelin associated glycoprotein (MAG) neuropathy. Schnitzler syndrome is a rare auto-inflammatory syndrome which is defined by monoclonal IgM deposition; in 15–20% of cases, Schnitzler syndrome progresses to WM [29]. Although the pathophysiology is not fully understood, it has been posited that aberrant inflammatory cytokine activation from interleukin-1 transcription due to *MYD88* L265P mutation, found ubiquitously in WM, is responsible for the mechanism of Schnitzler syndrome [30,31]. A defining criterion of Schnitzler syndrome is presence of an urticarial rash, stemming from the deposition of IgM along the dermal-epidermal junction. In many cases, standard treatment to WM and corticosteroids have limited therapeutic effect [32]. Anti-MAG neuropathy is closely associated with IgM gammopathy, as IgM binds to MAG to impair Schwann cell function and induce demyelination. It commonly presents with peripheral neuropathy: sensory loss and loss of vibration in extremities [33]. Similar to Bing–Neel syndrome, anti-MAG neuropathy may be treated with rituximab and/or bendamustine. When treated with rituximab monotherapy in a multicenter study, 44% of patients with anti-MAG secondary to WM saw overall improvement, with one case reporting significant improvement in compound muscle action potential in nerve conduction studies [34,35]. In one case diagnosed with both anti-MAG neuropathy and Bing–Neel syndrome, bendamustine monotherapy resolved sensory disturbance and weakness, improvement in nerve conduction studies, and normalized serum IgM levels [36]. When rituximab and bendamustine were combined in one case, serum IgM levels and anti-MAG titers improved proportionally [37].

## Conclusion

We present three cases of IgM monoclonal gammopathy with diagnosis of type 1 cryoglobulinemia, type 2 cryoglobulinemia, and rare presentation of WM with Bing–Neel syndrome. However, the wide breadth of clinical features of IgM monoclonal gammopathy and WM/LPL can make establishing both a diagnosis and optimal treatment selection difficult.
